# A Mutation in *VWA1*, Encoding von Willebrand Factor A Domain-Containing Protein 1, Is Associated With Hemifacial Microsomia

**DOI:** 10.3389/fcell.2020.571004

**Published:** 2020-09-09

**Authors:** Yibei Wang, Lu Ping, Xiaodong Luan, Yushan Chen, Xinmiao Fan, Lianyan Li, Yaping Liu, Pu Wang, Shuyang Zhang, Bo Zhang, Xiaowei Chen

**Affiliations:** ^1^Department of Otolaryngology, Peking Union Medical College Hospital, Chinese Academy of Medical Sciences and Peking Union Medical College, Beijing, China; ^2^Department of Otolaryngology, China-Japan Friendship Hospital, Beijing, China; ^3^Chinese Academy of Medical Sciences and Peking Union Medical College, Beijing, China; ^4^School of Medicine, Tsinghua University, Beijing, China; ^5^Department of Cardiology, Peking Union Medical College Hospital, Peking Union Medical College and Chinese Academy of Medical Sciences, Beijing, China; ^6^Tsinghua-Peking Center for Life Sciences, Tsinghua University, Beijing, China; ^7^Department of Otolaryngology, The Ohio State University, Columbus, OH, United States; ^8^Key Laboratory of Cell Proliferation and Differentiation of the Ministry of Education, College of Life Sciences, Peking University, Beijing, China; ^9^Department of Medical Genetics and National Laboratory of Medical Molecular Biology, Institute of Basic Medical Sciences, Chinese Academy of Medical Sciences and Peking Union Medical College, Beijing, China; ^10^Department of Otolaryngology Head and Neck Surgery, Beijing Tiantan Hospital, Capital Medical University, Beijing, China

**Keywords:** hemifacial microsomia, *VWA1*, zebrafish, cranial neural crest cell, whole exome sequencing

## Abstract

**Background:**

Hemifacial microsomia (HFM) is a type of rare congenital syndrome caused by developmental disorders of the first and second pharyngeal arches that occurs in one out of 5,600 live births. There are significant gaps in our knowledge of the pathogenic genes underlying this syndrome.

**Methods:**

Whole exome sequencing (WES) was performed on five patients, one asymptomatic carrier, and two marry-in members of a five-generation pedigree. Structure of WARP (product of *VWA1*) was predicted using the Phyre2 web portal. *In situ* hybridization and *vwa1*-knockdown/knockout studies in zebrafish using morpholino and CRISPR/Cas9 techniques were performed. Cartilage staining and immunofluorescence were carried out.

**Results:**

Through WES and a set of filtration, we identified a c.G905A:p.R302Q point mutation in a novel candidate pathogenic gene, *VWA1*. The Phyre2 web portal predicted alterations in secondary and tertiary structures of WARP, indicating changes in its function as well. Predictions of protein-to-protein interactions in five pathways related to craniofacial development revealed possible interactions with four proteins in the FGF pathway. Knockdown/knockout studies of the zebrafish revealed deformities of pharyngeal cartilage. A decrease of the proliferation of cranial neural crest cells (CNCCs) and alteration of the structure of pharyngeal chondrocytes were observed in the morphants as well.

**Conclusion:**

Our data suggest that a mutation in *VWA1* is functionally linked to HFM through suppression of CNCC proliferation and disruption of the organization of pharyngeal chondrocytes.

## Introduction

Hemifacial microsomia (HFM [MIM: 164210]), also known as otomandibular dysostosis and craniofacial microsomia, is a type of rare congenital genetic syndrome caused by developmental disorders of the first and second pharyngeal arches. The estimated occurrence rate is 1 in 5,600 live births, with no gender or laterality differences (Grabb, 1965; Xu et al., 2015). As one of the manifestations of the oculo-auriculo-vertebral spectrum (OAVS), recently re-estimated to show a prevalence of 3.8/100,000 live births, additional clinical findings are also frequently found in HFM patients, including other craniofacial anomalies and conditions affecting other organs and systems, such as urogenital anomalies, brain anomalies, microcephaly, heart defects, and short stature (Tasse et al., 2005). These variable clinical classifications have led to difficulties in classifying the severity of the disease. To date, several generations of classification systems have been developed, from the original Pruzansky-Kaban classification, which focuses mainly on the mandible and glenoid fossa (Kaban et al., 1988), to the SAT system, which takes skeletal malformations, auricular involvement and soft tissue defects into consideration, and finally to the widely accepted OMENS (Orbit, Mandible, Ear, Nerve, Soft tissue) and OMENS + system, which further includes additional disease manifestations (Tuin et al., 2015).

A complex etiology underlies this genetic syndrome. Environmental factors (e.g., retinoic acid exposure and vasoactive medications), maternal intrinsic factors (e.g., multiple pregnancies and maternal diabetes), and genetic factors, including both chromosomal variations (e.g., karyotype abnormalities of 14q22.3–q23.3) and mutations, can all lead to HFM (Ou et al., 2008; Northup et al., 2010). Most cases with documented pedigrees exhibit an autosomal-dominant inheritance pattern with incomplete penetrance and variable degrees of craniofacial deformities (Vendramini-Pittoli and Kokitsu-Nakata, 2009). To date, three possible pathogenic hypotheses have been proposed: (1) vascular abnormality and hemorrhage, which may interfere with the development of the first and second pharyngeal arches; (2) damage to Meckel’s cartilage, which may lead to apparent malformation; and (3) disruption of the development of cranial neural crest cells (CNCCs), which may disrupt the normal formation of maxillofacial bones (Chen et al., 2018). The three hypotheses are interconnected as CNCCs give rise to Meckel’s cartilage, and vascular abnormality can either interferes with CNCC development or cause damage to Meckel’s cartilage. Disruption of CNCC development can also in turn cause vascular malformation. Yet, there are significant gaps in our knowledge of the pathogenic genes underlying this syndrome.

In vertebrates, CNCCs origin from the ectoderm. They make up a mesenchymal core between the ectoderm and the endoderm along with the mesoderm cells and play important roles in the development of cartilage bones, peripheral nervous system and connective tissues of the head and neck (Schilling and Kimmel, 1994; Kimmel et al., 1998; Frisdal and Trainor, 2014). After gastrulation, CNCCs separate from the neural plate via epithelial-mesenchymal transition and migrate to the pharyngeal arches, where their development is further regulated by both intrinsic patterning information and endodermal signals such as RA (retinoic acid), FGF (fibroblast growth factor) and WNT (Frisdal and Trainor, 2014). In humans, the first pharyngeal arch gives rise to structures including the zygomatic arch, maxillary, mandible, malleus and incus, whereas the second pharyngeal arch gives rise to the stapes, styloid process and lesser horn of hyoid (Frisdal and Trainor, 2014). The patterning is conserved in zebrafish, where the first pharyngeal arch forms the jaw and the second forms jaw supporting structures (Schilling and Kimmel, 1994).

Here we analyzed an extended five-generation HFM pedigree. A novel candidate pathogenic gene, *VWA1* (MIM: 611901), encoding von Willebrand factor A domain-containing protein 1, was identified by whole-exome sequencing (WES). Functional studies in zebrafish showed that *vwa1* contributes to craniofacial cartilage development by supporting both the proliferation of CNCC and the organization of pharyngeal chondrocytes.

## Materials and Methods

### Participants

The study was conducted on an HFM pedigree at PUMCH (Peking Union Medical College Hospital). A comprehensive clinical history was taken and a complete physical examination was performed on all subjects. Ethical approval for this study was obtained from the institutional review board of PUMCH (JS-796). All participants or their legal guardians gave informed written consent to all clinical and genetic studies. All *in vivo* experiments were carried out in accordance with CALAS (Chinese Association for Laboratory Animal Science).

### WES and Variants Identification

WES was performed on five patients, one asymptomatic carrier, and two marry-in members of the pedigree. Genomic DNA was extracted from peripheral blood samples using a TIANamp Blood DNA Kit (Tiangen, Beijing, China) according to the manufacturer’s protocol. Exome enrichment was performed using a Sure Select Human All Exon v6 kit (65 Mb) (Agilent, Santa Clara, CA, United States), which yielded an average sequencing depth of 100-fold and a coverage of 99%. Uncovered regions were checked for large genomic deletions and none was found. Enriched shotgun libraries were sequenced on a HiseqX platform (Illumina, San Diego, CA, United States). SNPs and indels were functionally annotated using ANNOVAR and categorized into missense, nonsense and splice-site mutations, and other genomic features.

To identify the most likely pathogenic mutations, we filtered out (1) synonymous and non-coding variants (with the exception of splicing site mutations that might create an ectopic splicing site); (2) variants with an allele frequency of 0.005 or higher in the 1000 Genomes (1KG) Project, Exome Sequencing Project (ESP6500) and the Genome Aggregation Database (gnomAD); (3) variants that were not detected in one or more of the affected participants; and (4) variants that were present in the marry-in members of the pedigree. After filtration, rare or novel mutations in patients of the pedigree were verified by polymerase chain reaction (PCR) amplification and Sanger sequencing.

### Molecular Analysis of Variants

The webserver Phyre2^1^ and the online software program PSIPRED (v3.3)^2^ were used to predict secondary and tertiary structural variations associated with potentially pathogenic mutations of candidate genes (Huang et al., 2014; Kelley et al., 2015). The web-based program String^3^ was used to predict functional protein associations with WNT, BMP (bone morphogenic protein), FGF, RA, and endothelin-1 pathways, which are closely related to craniofacial development (Frisdal and Trainor, 2014; Graf et al., 2016).

### Zebrafish and Embryos

Adult zebrafish were maintained under standard conditions (Hans and Westerfield, 2007), and embryos were staged using standard methods (Kimmel et al., 1995). Tuebingen and the transgenic fish lines, *Tg (sox10:EGFP)* (ID:CZ156, ba2Tg/ +) and *Tg (nkx2.3:EGFP)* (Li et al., 2018), were used in the experiments.

### Knockdown and Knockout of *vwa1*

The morpholino oligonucleotide, 5′-TTC GGA CCT CCA TGA CGG GAC TAA A-3′, targeting the translation start site of zebrafish *vwa1* (*vwa1*-ATG MO) was designed and synthesized by Gene Tools (Gene Tools). To verify that the craniofacial deformities observed in these morphants were the result of reduced expression of *vwa1* rather than off-target effects of the morpholino, we injected a 1.5-fold (w/w) greater amount of a morpholino targeting *p53* (5′-GCG CCA TTG CTT TGC AAG AAT TG-3′) together with the *vwa1-*ATG MO (Robu et al., 2007). To validate the efficacy of *vwa1*-ATG MO, we constructed pCS2 + *vwa1*-ATG-MO-mCherry plasmid, inserting the morpholino binding sequence into the translation initiation site of mCherry. We injected the *in vitro* transcribed mRNA with/without *vwa1*-ATG morpholino into 1-cell embryos and observed the fluorescence at 1 dpf.

We also knocked out *vwa1* in zebrafish using the CRISPR/Cas9 system. Detailed methods were in accordance with previous work (Wu et al., 2018). Four guide RNAs (gRNAs, Supplementary Table 1) were mixed and co-injected with Cas9 into 1-cell stage embryos. The gRNAs were designed prior to construction of the Genome-Scale Guide Set using the CRISPR Design Website^4^. Templates for gRNA production were generated by annealing and elongating a forward primer containing a T7 promoter and guide sequence and a reverse primer encoding the standard chimeric gRNA scaffold. The sequences used are presented in Supplementary Table 1. The DNA template was *in vitro* transcribed, and gRNA was microinjected into 1-cell embryos. The efficacy of gRNA was verified by extracting crude genomic DNA from whole *vwa1*-knockout zebrafish embryos, followed by PCR amplification and sequencing (Wu et al., 2018). A morpholino targeting *upf3a* was also injected to inhibit compensatory effects of DNA damage (Shum et al., 2016).

### Whole-Mount *in situ* Hybridization

Whole-mount *in situ* hybridization (ISH) was performed on zebrafish as previously reported (Thisse and Thisse, 2008). A template for the *vwa1* antisense probe was constructed by amplifying full-length zebrafish *vwa1* using the forward primer 5′-ATG GAG GTC CGA AAG GCG CT-3′ and the reverse primer 5′-TAA TAC GAC TCA CTA TAG GGT CAA GCC GTG CAG CAG AGT C-3′ containing the T7 polymerase sequence. The antisense probe was synthesized using T7 polymerase. Probes against *dlx2a* (Akimenko et al., 1994), *crestin* (Xia et al., 2013), and *sox9a* (Chiang et al., 2001) were also used. Unless stated otherwise, these experiments were repeated for three times, and at each time 30 embryos were stained for each gene at each experimental setting.

### Cartilage Staining

Embryos were fixed in 4% paraformaldehyde (PFA) at 4°C overnight and then washed in phosphate-buffered saline containing 0.1% Tween-20 (PBST) for 8 h. After staining cartilage with 0.015% Alcian blue overnight at 4°C, embryos were rehydrated with a graded series of alcohols to distilled water and then treated with 0.25% trypsin at room temperature until embryo tissues became transparent. The embryos were then transferred to a solution of 1% KOH/3% H_2_O_2_ for 10 min and washed twice in PBST for 5 min each (Li et al., 2018). Embryos were also fixed and stained with Wheat Germ Agglutinin (WGA) (Invitrogen) and DAPI (Sigma-Aldrich) as described by Dale and Topczewski (2011) to show the structure of pharyngeal chondrocytes (Sisson et al., 2015). Z-stack images of the embryos were taken using either a Zeiss LSM 510 or LSM 700 laser confocal microscope. Unless stated otherwise, these experiments were repeated for 3 times, and at each time 50 embryos were stained for each injection setting.

### Immunofluorescence

*Tg (sox10:EGFP)* zebrafish embryos were fixed with 4% phosphate-buffered paraformaldehyde and washed with PBST for 20 min, then immunostained with PHH3 (1/400; sc-374669, Santa Cruz Biotechnology) antibodies. Terminal deoxynucleotidyl transferase dUTP nick-end labeling (TUNEL) assays were performed using the *In Situ* Cell Death Detection Kit, TMR red (12156792910, Roche) according to the manufacturer’s instructions. DAPI (4′,6-diamidino-2-phenylindole) was used to visualize nuclei. Anti-PHH3 staining was designed to indicate proliferation and TUNAL assays to indicate apoptosis. In either analysis, only cells that were double positive for anti-PHH3/TUNEL and EGFP were counted manually in order to make sure that they were CNCCs. All immunofluorescence images were captured using a Nikon A1R + confocal microscope using the same settings for all experiments. The experiments were repeated for 2 times.

### Statistical Analysis

All experiments were performed in triplicate, and unpaired *t*-tests were used to analyze all data sets. Results were considered statistically significant at *p* < 0.05. Congruent results (>85% of the embryos observed) were reached in all phenotypic observational experiments and all selected images are representative.

## Results

### Clinical Presentation

**FIGURE 1:**
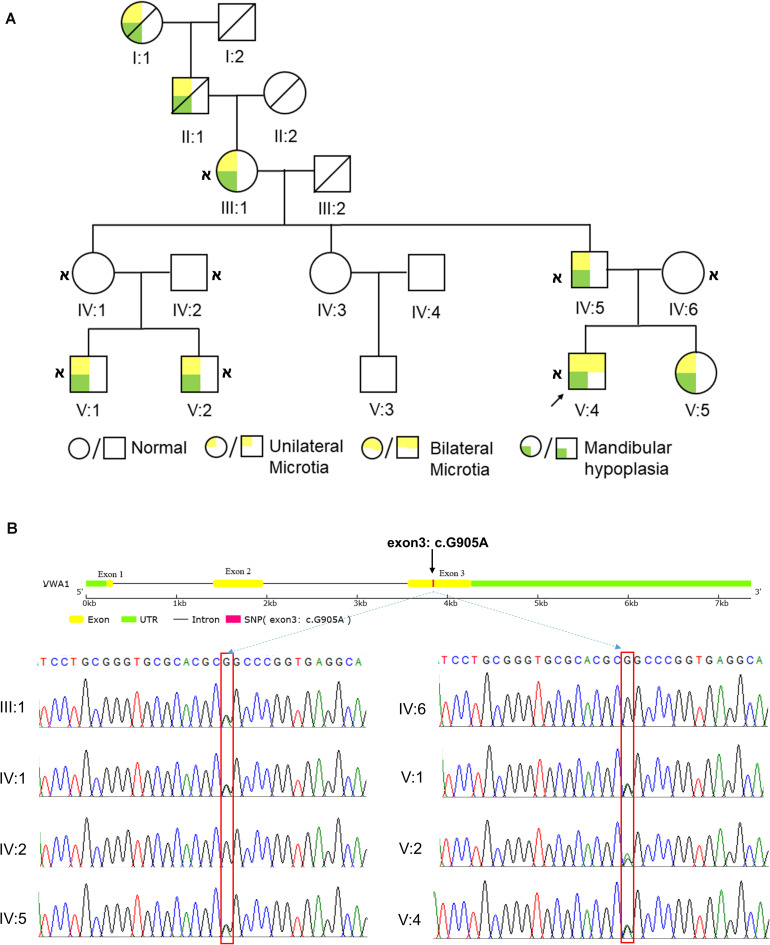
HFM pedigree and the identification of *vwa1*. **(A)** Diagram of the five-generation HFM pedigree. This is a five-generation Chinese kinship in which 8 of 11 direct relatives displayed HFM to varying degrees. The proband showed grade III bilateral ear deformities; individual III:1 in the pedigree had a grade II unilateral ear deformity, whereas other patients exhibited unilateral grade III ear deformities. All patients exhibited mandibular hypoplasia. Circles denote females and squares denote males. The text immediately below the symbols shows the ID of the corresponding family member; 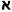 indicates that whole-exome sequencing was done. **(B)** Sanger sequencing results of the *VWA1* mutation in the pedigree. Patients III:1, IV:5, V:1, V:2 and V:4, and the asymptomatic carrier IV:1, all had the same non-synonymous point mutation (c.G905A:p.R302Q) in *VWA1*, whereas the two marry-in members of the family (IV:2 and IV:6) did not.

8 of 11 direct relatives in this five-generation pedigree displayed HFM to varying degrees (Figure 1A). The proband showed grade III bilateral ear deformities and III:1 in the pedigree had grade II unilateral ear deformity according to the Marx classification, whereas other patients exhibited unilateral grade III ear deformities. All patients exhibited mandibular hypoplasia. Pure-tone audiometry testing showed moderate to severe conductive hearing loss. The family denied consanguinity. Detailed physical examination, along with cardiac, renal, and abdominal ultrasound was carried out on all 12 living members of the pedigree and no deformities of other systems were diagnosed. The disorder appeared to follow an autosomal-dominant segregation pattern with incomplete penetrance and variable expressivity. Individual IV:3 and V:3 showed no malformation of all the systems examined, and refused to give blood for sequencing. Individual IV:1 was predicted to be a carrier of the pathogenic gene.

### Identification *of VWA1* Mutation and Molecular Analysis Results

In order to identify the variants, we first collected peripheral blood from family members and extracted cDNA. Individual IV:1 was regarded as carrier and the two marry-in members (IV:2 and IV:6) were regarded as controls. After careful filtration as mentioned in the method section, WES revealed eight candidate genes: *VWA1*, *SLC35G4*, *HRC*, *KLK1*, *ZBTB45*, *FBLN2*, *HECW1*, and *PGBD3*. These eight genes were present in all six living affected individuals tested. All candidate genes went through secondary and tertiary structure predictions and expression profiles of all genes that have homologs in zebrafish were constructed (data not shown). Basing on: (1) *VWA1* mutation caused the most apparent structural changes; (2) *VWA1* associated variance had previously been reported in a patient presenting with mandibulofacial dysostosis and microtia (Zhang et al., 2010); (3) *vwa1* showed specific expression in the mandible area of zebrafish, we suspected that *VWA1*, encoding WARP (von Willebrand factor A-domain-related protein 1), was most likely associated with HFM. The non-synonymous point mutation (c.G905A:p.R302Q) in the *VWA1* gene was shared by all patients tested and the predicted carrier as well (Figure 1B).

**FIGURE 2:**
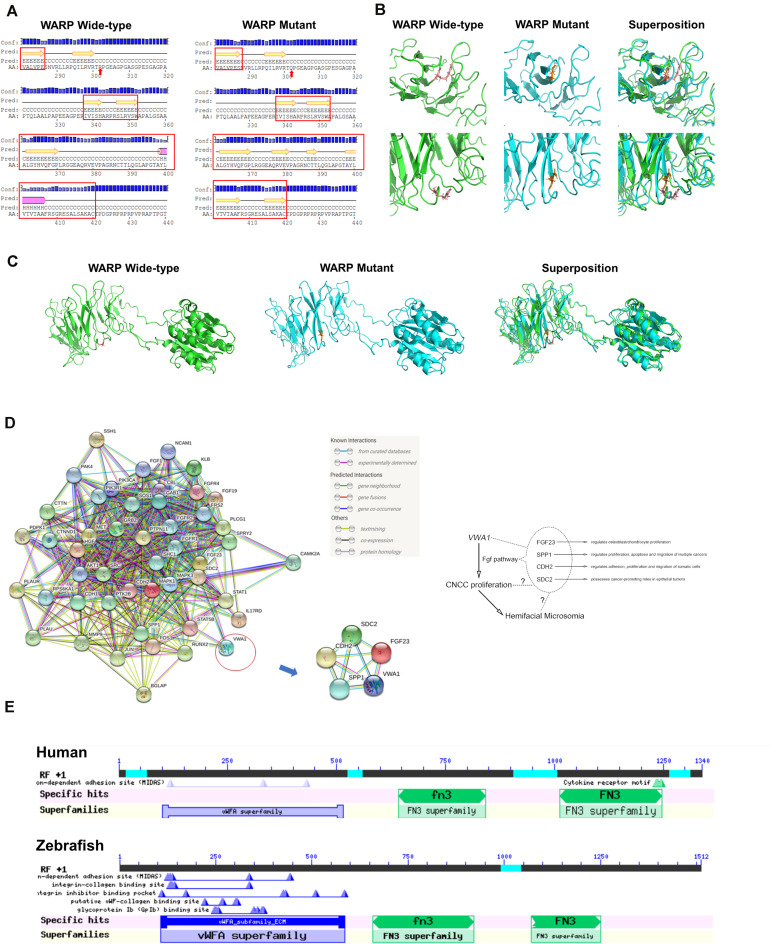
Predicted WARP structures, protein interactions and conserved domains. **(A)** Predicted secondary structures of wild-type and mutant protein sequences flanking the mutation. Secondary structural features are annotated as follows: pink cylinder, α-helix; yellow arrow, β-sheet; black line, coil; Conf, confidence; Pred, predicted; H in Pred line, helix; C in Pred line, coil; E in Pred line, sheet; AA, amino acid; red arrow, mutant amino acid. Local **(B)** and global **(C)** views of the predicted tertiary structures of wild-type (green) and mutant (blue) proteins. Structural superposition analyses demonstrating the change in protein structure brought about by the mutation are also displayed. **(D)** Protein-to-protein interactions of WARP with the FGF pathway. Predictions using the web-based program, String, indicated that WARP may functionally interact with four proteins of FGF pathway: FGF23, SPP1, CDH2, and SDC2. **(E)** Domains of WARP conserved in humans and zebrafish. Humans and zebrafish have the same WARP conserved domains: a von Willebrand factor A-domain, the first fibronectin type III repeat, and the second fibronectin type III repeat.

Secondary and tertiary structures of sequences flanking the mutation were predicted (Figures 2A–C). The mutation introduced a R302Q amino acid substitution in the protein’s loop area, altering upstream and downstream secondary structures as well, including the possible disruption of a helix and introduction of several sheets (Figure 2A). The change from arginine, a basic amino acid, to glutamine, a neutral amino acid, altered the position of the amino acid’s side chain and brought changes to the local tertiary structural prediction, indicating changes in both the conformation and function of WARP (Figures 2B,C). Prediction of protein-to-protein interactions of WARP were carried out and five pathways: WNT, BMP (bone morphogenic protein), FGF, RA, and endothelin-1, which are closely related to craniofacial development, were selected for detailed analysis (Frisdal and Trainor, 2014; Graf et al., 2016). This analysis showed that WARP may interact with four proteins-FGF23, SPP1 (osteopontin), CDH2 (cadherin 2), and SDC2 (syndecan 2)-of the FGF pathway (Figure 2D). These taken together indicated that *VWA1* is indeed a strong candidate pathogenic gene of this HFM pedigree.

### *VWA1* Homolog in Zebrafish, *vwa1*, Is Specifically Expressed in the Mandible

The protein encoded by *VWA1* in humans was shown to contain three conserved domains: a von Willebrand factor A-domain, the first fibronectin type III repeat and the second fibronectin type III repeat. There is one homologous gene, *vwa1*, in zebrafish which has the same conserved domains (Figure 2E).

**FIGURE 3:**
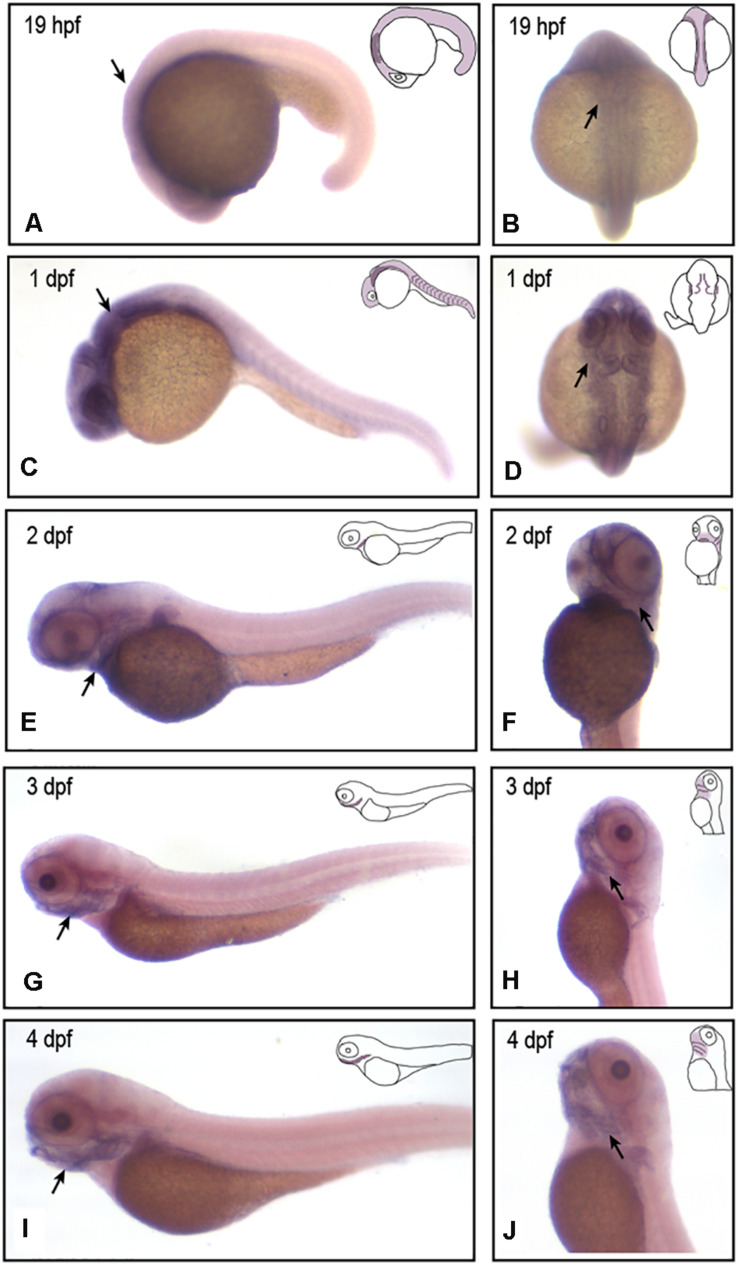
*vwa1* expression in developing zebrafish embryos. *In situ* hybridization (ISH) results showing the expression of *vwa1* at 19 hpf **(A,B)**, 1 dpf **(C,D**), 2 dpf **(E,F)**, 3 dpf **(G,H)** and 4 dpf **(I,J)** stages in lateral **(A,C,E,G,I),** dorsal **(B,D**) and lateral-ventral **(F,H,J)** views. At 19 hpf and 1 dpf, *vwa1* was expressed throughout the body, with expression in the head being higher than that in other parts of the body (black arrows in **A,C**). At 19 hpf and 1 dpf, *vwa1* expression was detected at the medial edge of the pharyngeal arch (black arrows in **B,D**). At 2 dpf, *vwa1* staining in the head began to increase slightly, especially in the mandible (black arrow in **E**). At 3 dpf and 4 dpf, expression of *vwa1* was restricted to the mandible (black arrows in **G,I**). Staining of *vwa1* in the pharyngeal pouch was stronger than that in the pharyngeal arch (black arrows in **F–H**). *vwa1* was also expressed in the epidermis of the brain **(D)** and in somites **(C,E,G,I)**.

To verify that *vwa1* plays an important role in craniofacial development, we first examined its spatiotemporal expression during zebrafish embryonic development using *in situ* hybridization (*n* = 45). We found that, from 19 h post-fertilization (hpf) to 1 day post-fertilization (dpf), *vwa1* expression was observed at the medial edge of the pharyngeal arch, with a slight concentration in the rostral region that gives rise to craniofacial structures (Figures 3A–D). At 19 hpf and 1 dpf (Figures 3B,D). From 2 to 4 dpf, expression of the *vwa1* gene in the head became increasingly concentrated in the mandibular region (Figures 3E–J). Staining for *vwa1* in the pharyngeal-pouch was more intense than that in the pharyngeal arch (Figures 3F–H). Expression of *vwa1* was also found in the epidermis of the brain and somites (Figures 3C,D), which was in accordance with previous reports in mice (Brachvogel et al., 2008; Allen et al., 2009). These observations strongly suggest that zebrafish *vwa1* is specifically expressed in the mandible during the period of pharyngeal arch development.

### Reduced *vwa1* Expression Causes Malformation of Pharyngeal Cartilage

To determine if *vwa1* is required for the development of pharyngeal cartilages, we knocked down its function in zebrafish embryos using an antisense morpholino oligonucleotide targeting the translation start site of *vwa1* mRNA (*vwa1*-ATG MO) at the 1-cell stage, which is proved effective. Higher amounts of *vwa1*-ATG MO caused more severe deformities (Supplementary Figure 2), and in subsequent experiments a dose of 1 ng was applied.

**FIGURE 4:**
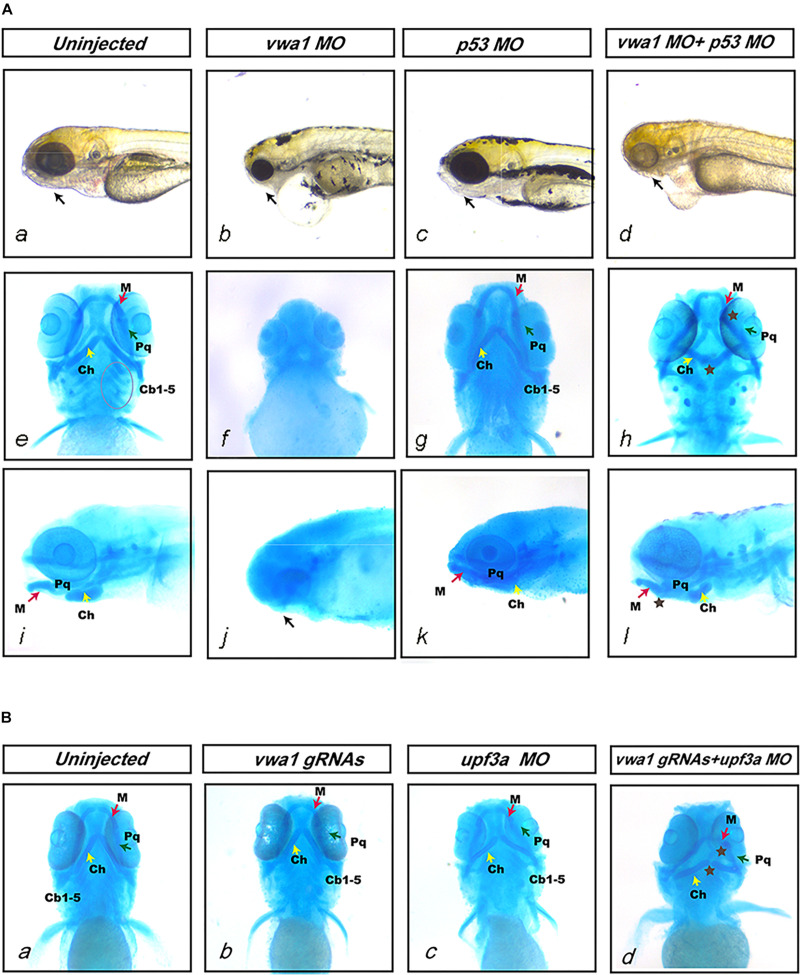
Deformities of pharyngeal cartilage in *vwa1* morphants and mutants. **(A)** Deformities of pharyngeal cartilage in *vwa1* morphants. The figure shows the jaws of uninjected and *vwa1* morpholino (MO)-, *p53* MO- and *vwa1* + *p53* MO-injected morphants under a stereoscope and Alcian blue staining of morphant embryos. Injection of 1 ng *vwa1*-ATG MO caused jaw structures to almost completely disappear (b, f, and g). Injection of 1 ng *vwa1*-ATG MO with *p53* MO reduced the overall size of jaw structures at 4 dpf compared with uninjected controls and 1.5 ng *p53* MO-injected morphants (a and d), whereas there was no significant difference compared with controls after injection of *p53* MO (a–d and i–l). The first and second pharyngeal arches were severely hypoplastic (brown ⋆ in h and l), and the third to seventh pharyngeal arches (Cb1-5, circled in e) were absent (h). Joint junctions were abnormal: the angle between cartilage derived from the first pharyngeal arch, Meckel’s (M) and palatoquadrate (Pq) became smaller, whereas the angle between cartilage derived from the second pharyngeal arch ceratohyal (Ch) became larger (brown ⋆ in h and l). **(B)** Deformities of pharyngeal cartilage in *vwa1*-knockout mutants. a, b, c, and d shows Alcian blue staining results for uninjected embryos and embryos injected with *vwa1* gRNAs, *upf3a* MO, and *vwa1* gRNAs together with *upf3a* MO. Embryos injected with *vwa1* gRNAs or *upf3a* MO showed no significant deformities in pharyngeal cartilage compared with uninjected embryos (b and c). Injection of *vwa1* gRNAs together with *upf3a* MO resulted in pharyngeal cartilage deformities similar to those in *vwa1* morphants, including hypoplasia of first and second pharyngeal cartilages, disappearance of the third to seventh pharyngeal arches, and abnormal joint junctions (brown ⋆ in d).

To reduce off-targeting effects of morpholinos, we co-injected a 1.5-fold (w/w) greater amount of *p53* MO together with *vwa1*-ATG MO (Robu et al., 2007). Previous research has shown that *p53* null mutation in mice can rescue the craniofacial damage caused by disruption of *Brca1* (Robu et al., 2007; Kitami et al., 2018). We performed injection of *p53* at different concentration without *vwa1*-ATG MO to rule out its potential effects and no visible malformation was observed at each concentration (Supplementary Figure 2). Co-injection of *p53* MO together with *vwa1*-ATG MO induced observable phenotypic changes in 132/150 embryos. After injecting 1 ng *vwa1*-ATG MO and 1.5 ng *p53* MO, there was a reduction in the overall size of jaw structures at 4 dpf compared with uninjected controls (Figure 4Aa,d). Alcian blue staining revealed that the first and second pharyngeal arches were severely hypoplastic, and the third to seventh pharyngeal arches (Cb1-5) were absent (Figure 4Ae,h). The joint junctions were also abnormal: the angle between cartilage derived from the first pharyngeal arch, Meckel’s (M) and palatoquadrate (Pq), became smaller, and the angle between cartilage derived from the second pharyngeal arch, ceratohyal (Ch), became larger (Figure 4Ae,i,h,l). Besides, injected embryos also demonstrated ventral swelling suggestive of cardiovascular dysfunction. Whether *vwa1* had potential effects on cardiovascular development would require further study.

To further verify the causal relationship between *vwa1* variants and the malformed pharyngeal cartilages observed in *vwa1* zebrafish mutants, we knocked out *vwa1* using four different gRNAs, all of which proved effective. Alcian blue staining revealed no deformities in the *vwa1-*knockout mutant (Figure 4Bb). Given the phenotype of *vwa1*-ATG morphants, this unexpected finding suggested potential compensatory effects. To mitigate potential genetic compensatory effects attributable to DNA damage, we co-injected *vwa1-*knockout mutants with morpholinos targeting *upf3a* to inhibit nonsense-mediated RNA decay (Shum et al., 2016). Embryos injected only with *upf3a* MO had no significant deformities in pharyngeal cartilages (Figure 4Bc). Embryos co-injected with *upf3a* MO showed deformities in pharyngeal cartilage similar to those of *vwa1*-ATG morphants, including hypoplasticity of the first and second pharyngeal cartilages, the disappearance of third to seventh pharyngeal arches and abnormal joint junctions (Figure 4Bd). Collectively, these data suggest that *vwa1* plays a very important role in craniofacial development, such that its loss causes deformities of pharyngeal cartilage.

### Migration and Differentiation of CNCCs Are Not Affected by *vwa1*

**FIGURE 5:**
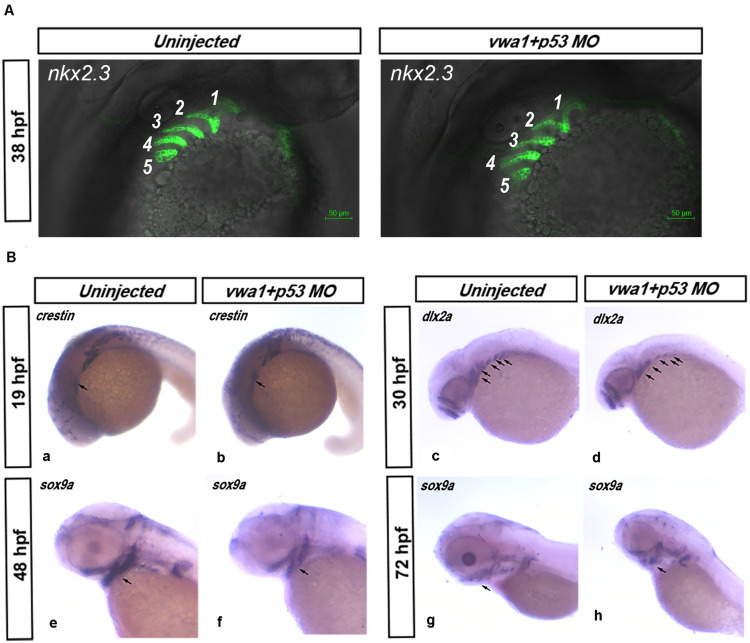
Effects of reduced *vwa1* on the development of pharyngeal cartilage. **(A)** Endodermal pouch in *Tg (nkx2.3:EGFP)* embryos. At about 38 hpf, when the endodermal pouch is almost fully formed, no apparent difference was observed in the pharyngeal pouch in *vwa1* morphants (right) compared with uninjected morphants (left). **(B)** *vwa1* knockdown causes a reduction in cranial neural crest cell numbers. At about 19 hpf, when neural crest cells migrate to the cranial area, the expression of *crestin* in *vwa1*-knockdown embryos was not obviously different from that of uninjected control embryos (a and b). At 30 hpf, when migration of neural crest cells is complete, *dlx2a* was expressed in similar domains, but the area of these domains was reduced significantly in *vwa1* morphants (c and d). At 48 and 72 hpf, when the cranial neural crest has differentiated into pharyngeal chondrocytes, *sox9a* was expressed in similar domains, but the areas of these domains were reduced significantly in *vwa1* morphants (e–h). These data show that, in *vwa1* morphants, neural crest cells generally could migrate to the pharyngeal arch and specify to chondrocytes, but their numbers were reduced.

The higher expression of *vwa1* in the pharyngeal endoderm led us to examine morphogenesis of the pharyngeal pouch in *vwa1*-ATG morphants. To this end, we knocked down the expression of the *vwa1* in *Tg (nkx2.3:EGFP)* transgenic fish (Li et al., 2018), in which we could observe the pharyngeal endoderm cells. At approximately 38 hpf, the pharyngeal pouch in *vwa1*-ATG morphants appeared somewhat wider, but there were no striking differences compared with controls (Figure 5A).

We next used *in situ* hybridization to monitor the expression of the following marker genes involved in the migration and specification of the CNCCs: *crestin*, which is expressed in neural crest at about 19 hpf, a time when CNCCs migrate to the cranial area; *dlx2a*, which is expressed in all CNCCs in the pharyngeal arches at 30 hpf, when CNCCs have completed their migration; as well as *sox9a*, markers of cartilage differentiation (Yan et al., 2002; Johnson et al., 2011; Kim et al., 2015). At 19 hpf, there was no obvious difference in *crestin* expression between *vwa1*-ATG morphants and uninjected embryos, suggesting that CNCCs can migrate normally to the arches. At 30, 48 and 72 hpf, *dlx2a* and *sox9a* were expressed in similar domains, but the areas of these domains were significantly reduced in the *vwa1*-ATG morphants compared with uninjected embryos. These data show that, in the *vwa1*-ATG morphants, CNCCs were generally capable of migrating to the pharyngeal arches and differentiating into chondrocytes, but their number was reduced (Figure 5B).

### Proliferation of CNCCs Requires *vwa1*

**FIGURE 6:**
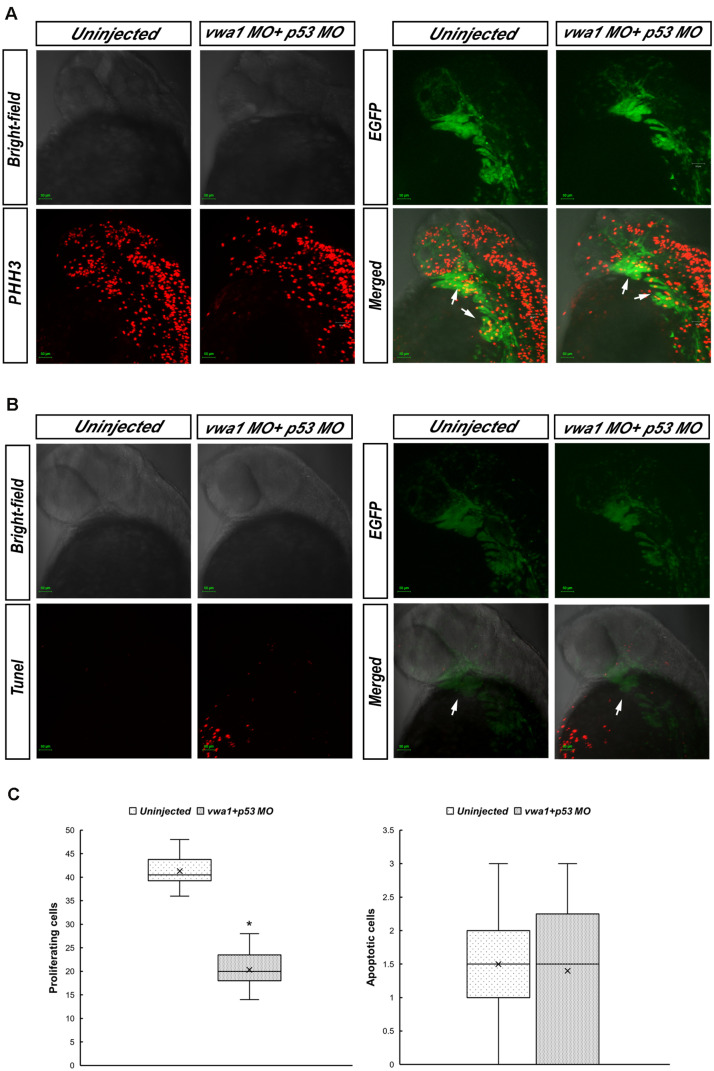
Proliferation and apoptosis of cranial neural crest cells at approximately 30 hpf. **(A)** Immunofluorescence results showing anti-phosphohistone H3 (PHH3) staining demonstrating cell proliferation. Merged image demonstrates higher anti-PHH3 signals in cranial neural crest cells compared with uninjected embryos. Arrows: pharyngeal arches. **(B)** Apoptosis assessed by immunofluorescence detection of TUNEL assay. The apoptosis signal in the *vwa1* + *p53* MO group did not coincide with EGFP fluorescence. Arrows: pharyngeal arches. **(C)** Comparison of apoptotic and proliferative numbers of neural crest cells in pharyngeal arches between *vwa1* + *p53* MO-injected embryos and uninjected control embryos. The difference in cell proliferation was statistically significant (*p* < 0.05, Student’s *t*-test). Lower/upper whiskers: 1^st^ and 4^th^ quartiles of the data; box: 2^nd^ and 3^rd^ quartiles of the data; mid-line: median of the data; ×: mean value of the data; *: significantly different.

We hypothesized that the reduction in the number of CNCCs was attributable to changes in their proliferation and/or apoptosis. To identify proliferating and apoptotic cells, we immunostained for PHH3 and performed TUNEL assay, respectively, at 30 hpf. To confirm that the cells we observed in pharyngeal arches were indeed CNCCs, we used *Tg (sox10:EGFP)* embryos. Immunofluorescence images were captured (Figures 6A,B) and statistical analysis of 20 *vwa1*-ATG morphants vs. 20 uninjected embryos on cell proliferation, and 10 *vwa1*-ATG morphants vs. 10 uninjected embryos on cell apoptosis showed that proliferation of CNCCs was reduced significantly from an average of 41.30 ± 3.02 cells per fish (uninjected embryos) to 20.35 ± 3.45 cells per fish (*vwa1*-ATG morphants) (*n* = 20, *p* < 0.05), whereas apoptosis was not significantly affected, with that of that of uninjected embryos being 1.50 ± 0.81 cells per fish and *vwa1*-ATG morphants being 1.40 ± 1.11 cells per fish (*n* = 10). These data, combined with the bioinformatics prediction of protein-to-protein interactions of WARP (Figure 2D), suggest that *vwa1* is required for the proliferation of CNCCs. It may regulate CNCC proliferation via crosstalk with FGF pathway and its knockdown leads to deformities of pharyngeal cartilages by reducting CNCC proliferation.

### *vwa1*-ATG Morphants Exhibit Disorganization of Pharyngeal Chondrocytes

**FIGURE 7:**
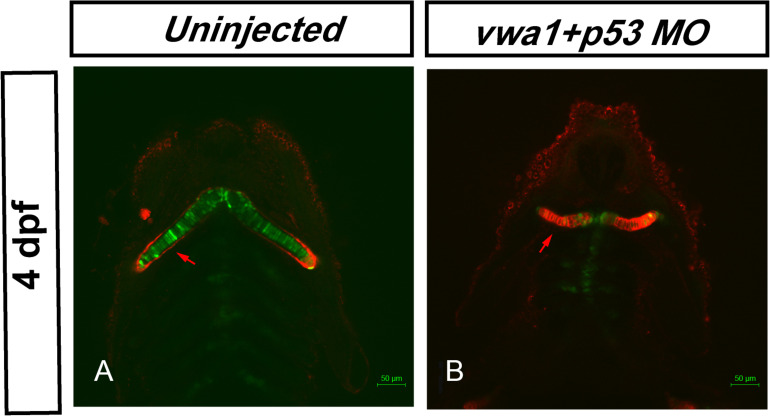
Knockdown of *vwa1* leads to disorganization of pharyngeal chondrocytes at 4 dpf. **(A)** In uninjected control embryos at 4 dpf, craniofacial cartilage had formed and showed a very stereotypic shape between individuals, exhibiting the characteristic “stack of pennies” organization in which thin and elongated chondrocytes are assembled on top of one another to form their respective cartilage element. **(B)** In *vwa1* morphants, the size and length-width ratio of many cartilage chondrocytes were smaller and the cartilage elements were greatly deformed compared with those of uninjected controls.

As WARP is localized to the extracellular matrix of chondrocytes (Allen et al., 2006), we hypothesized that the arrangement of chondrocytes might be altered and could contribute to the pharyngeal cartilage deformities. To examine this, we used fluorescent wheat germ agglutinin (WGA) to stain glycoproteins in the chondrocyte membrane. The uninjected embryos exhibited a very stereotypic shape among individuals at 4 dpf, with thin and elongated chondrocytes assembled on top of one another, forming a characteristic “stack of pennies” organization (Figure 7A). In the *vwa1*-ATG morphants, many of the cartilage elements were not only smaller, but greatly deformed as well-compared to the uninjected ones (Figure 7B). These results show that knockdown of *vwa1* leads to disorganization of pharyngeal chondrocytes and subsequent deformities in pharyngeal cartilage.

## Discussion

In the present study, point mutations in 8 candidate genes shared by all affected individuals: *VWA1*, *SLC35G4*, *HRC*, *KLK1*, *ZBTB45*, *FBLN2*, *HECW1* and *PGBD3*, were identified by WES in this five-generation HFM pedigree. Basing on structural prediction, literature review and functional study in zebrafish, we predicted the c.G905A:p.R302Q mutation in the *VWA1* gene to be most likely associated with HFM. In the fifth generation, the mutation in V:1 and V:2 was inherited from a non-HFM mother (IV:1), indicating incomplete penetrance of the disease. Although only a single base is mutated, *in silico* prediction demonstrated that it may lead to significant changes in the secondary and tertiary structure of *VWA1* protein, suggesting substantial changes in protein function.

*VWA1* was primarily known for its expression in cartilage cells, in basement membranes of the peripheral nervous system and in skeletal and cardiac muscle (Fitzgerald et al., 2002; Allen et al., 2008; Fitzgerald, 2019). Subsequent research also revealed a distinct localization of *VWA1* in the inner ear vasculature, suggesting a potential role in maintaining its integrity (Duong et al., 2011). Although *VWA1* has surfaced in research on several diseases and has been suspected to be associated with neuro-musculoskeletal disorders, to date, no direct disease-causing variants have been directly identified (Dieppedale et al., 2013; Fitzgerald, 2019). Zhang et al. (2010) previously reported copy number variance of *VWA1* and *PYGO2* in a patient presenting with mandibulofacial dysostosis and microtia. At that time, *VWA1* was suspected, but not confirmed, to be among the candidate genes responsible for the abnormal phenotype. Considering the low incidence of HFM, the fact that the mutation in was found in unrelated sporadic case and a five-generation pedigree reinforces the validity of the conclusion that the c.G905A:p.R302Q mutation in *VWA1* is a likely pathogenic mutation for HFM.

Knockdown of *vwa1* using *vwa1*-ATG MO resulted in hypoplasia of facial cartilage in zebrafish. In contrast, knockout of *vwa1* using CRISPR/Cas9 (with four different gRNAs) did not cause deformities in pharyngeal cartilage. Notably, *Vwa1* knockout in mice also did not induce observable craniofacial deformities as was reported in a previous study (Allen et al., 2009). The inconsistency in *vwa1*-ATG MO knockdown and CRISPR/Cas9 knockout led us to further inspect the mechanisms behind. We speculated that the lack of pharyngeal cartilage deformity in *vwa1*-knockout zebrafish was attributable to genetic compensation, which is considered a possible explanation for phenotypic differences between gene knockout and gene knockdown (Ma et al., 2019). mRNA bearing a premature termination codon promptly triggers a genetic compensation response that involves *upf3a* and components of the COMPASS complex (Ma et al., 2019). After we knocked down the function of *upf3a*, the *vwa1*-knockout zebrafish exhibited deformities similar to those in *vwa1*-knockdown embryos. These data show that *vwa1* dysfunction genuinely affects craniofacial development. Notably, apart from craniofacial anomalies, knockdown of *vwa1* also resulted in ventral swelling, which might indicate cardiovascular dysfunction. Neural crest cells also participate in the development of the heart and similar situation where a gene plays important roles in both ear and cardiovascular development has been reported in *eya4* (Schönberger et al., 2005; Wentzel and Eriksson, 2011). Therefore, whether *vwa1* holds potential effects on cardiac development requires further exploration. The phenotypic changes in zebrafish indicated potential haploinsufficiency mechanisms behind the potential pathogenic effects of *vwa1*. However, the possibility that truncated WARP produced by the point mutation in humans may interfere with normal WARP function and demonstrate a dominant negative pathogenic fashion was not excluded (Messina et al., 2016a, b).

In *vwa1*-ATG morphants, the chondrocytes of pharyngeal cartilage appeared smaller and disorganized compared with the normal characteristic “stack of pennies” organization. This observation is understandable as WARP serves as an important component of the extracellular matrix (ECM), whose composition is a complex mixture of collagens and non-collagenous proteins (Allen et al., 2006). Research has revealed a regulatory role for proteoglycans, glycosaminoglycans, proteases, and glycosidases in cell proliferation and differentiation (Roughley, 2006). Moreover, the special avascular nature of cartilage further highlights the important effects of the ECM on the diffusion, distribution, and binding capacity of signaling molecules (Esko and Selleck, 2002; Cortes et al., 2009). ECM proteins also have the capacity to bind to receptors and initiate signal transduction (Streuli, 1999). Therefore, a disturbance in the ECM microenvironment caused by knockdown of *vwa1* could contribute to the overall phenotypic deformities in pharyngeal cartilage.

Upon reducing *vwa1* function, the remaining cartilaginous elements in *vwa1*-ATG morphants seemed to adopt appropriate patterns, both the first and second pharyngeal arch derived structures were affected, and markers for craniofacial development were generally expressed in appropriate locations. These data suggest that *vwa1* does not play a vital role in the migration, aggregation or specification of cranial neural crest cells, but may be very important in regulating their number. In support of this hypothesis, the size of both craniofacial cartilage and cranial neural crest cell marker domains were reduced in *vwa1*-ATG morphants, indicating a change in CNCC number. Reduced CNCC number could result from decreased proliferation and increased apoptosis, which have both been proven to be associated with different kinds of craniofacial anomalies. For example, reduced proliferation of CNCCs in the medial nasal process mesenchyme is required for upper lip closure and has been identified in the pathogenesis of cleft lip (Everson et al., 2017). Meanwhile, increased apoptosis of CNCCs could affect secondary palate fusion and has been proven to be associated with significant mid-facial anomalies such as cleft palate (Wilson et al., 2016; Welsh et al., 2018; Wang et al., 2019). Based on our data, we observed a decrease in cell proliferation, but no change in cell apoptosis, in *vwa1*-ATG morphants. Taken together, these data indicate that *vwa1* is required for the proliferation of CNCCs.

CNCCs’ proliferation, apoptosis, migration and differentiation have been reported to be tightly regulated by a delicate and intricate extracellular signaling system, which includes SHH, WNT, RA, FGF, endothelin and BMP (Frisdal and Trainor, 2014; Graf et al., 2016; Everson et al., 2017). As an important component of the ECM, *VWA1* is likely to regulate CNCC proliferation via extracellular signal transduction. Bioinformatics analyses predicted possible crosstalk of WARP with four proteins in the FGF pathway: FGF23, SPP1, CDH2, and SDC2. The FGF pathway is known to play crucial roles in the specification, patterning and migration of cranial neural crest cells (Diez Del Corral and Morales, 2017). Elements of this pathway have also been shown to regulate cranial neural crest cell proliferation (Sasaki et al., 2006). In particular, FGF23, a bone-derived hormone produced by osteoblasts and osteocytes that signals through FGFRs and α-Klotho, has a well-established association with renal phosphate wasting diseases (Erben, 2017). While research has demonstrated regulatory roles of FGF23 on osteoblast and chondrocyte proliferation *in vitro* and *in vivo* (Kawai et al., 2013), its effects on cranial neural crest cells await further study. CDH2 plays important roles in the adhesion, proliferation and migration of somatic cells (Marie et al., 2014), and has also been reported to function in the morphogenesis of zebrafish inner ear (Babb-Clendenon et al., 2006). SPP1 is an ECM protein closely associated with tumorigenesis and bone remodeling (Sousa et al., 2012; Saleh et al., 2016). Previous researches have reported its potential expression in cranial suture tissues of zebrafish (Topczewska et al., 2016). SDC2 has been reported to regulate angiogenic sprouting in zebrafish (Chen et al., 2004), and has distinct expression in embryonic zebrafish brain (Hofmeister et al., 2013). The overlap of spatiotemporal expression patterns and functional similarities between WARP and these proteins further support the possibility of their potential interactions. The crosstalk with these membrane proteins and secreted signaling molecules suggests complicated interactions among signaling pathways, especially the FGF pathway, which may underlie the suppressive effects of *vwa1* knockdown on the proliferation of cranial neural crest cells (Figure 2D).

## Conclusion

We reported that *VWA1* is a strong candidate gene for HFM, showing that *vwa1* variants influences the proliferation of CNCCs and the organization of pharyngeal chondrocytes. More patients with *VWA1* mutations and corresponding genetic mouse models will be required to further confirm the causal role of this gene. The identification of this candidate gene for HFM can not only help to decipher the pathways involved in craniofacial development, but provide possible targets for genetic testing and therapeutic intervention as well.

## Data Availability Statement

The raw data supporting the conclusions of this article will be made available by the authors, without undue reservation.

## Ethics Statement

The studies involving human participants were reviewed and approved by the institutional review board of PUMCH (JS-796). Written informed consent to participate in this study was provided by the participants’ legal guardian/next of kin. The animal study was reviewed and approved by the institutional review board of PUMCH (JS-796).

## Author Contributions

XC and SZ obtained funding. YW, SZ, and XC conceived the study. YW and BZ designed the experiments. YW and LL conducted the experiments. YW and LP participated in the literature search, data collection, data analysis, and drafted the manuscript. XL conducted the bioinformatics protein structural prediction. YC, XF, and PW participated in the collection and treatment of the pedigree. YL participated in the genetic analysis of the pedigree. All authors have read and approved the final manuscript.

## Conflict of Interest

The authors declare that the research was conducted in the absence of any commercial or financial relationships that could be construed as a potential conflict of interest.

## Footnotes

1 http://www.sbg.bio.ic.ac.uk/phyre2

2 http://bioinf.cs.ucl.ac.uk/psipred/

3 https://string-db.org

4 http://crispr.mit.edu

## Supplementary Material

FIGURE S1 Efficacy of *vwa1*-ATG Morpholino. **(A)** Structure of pCS2 + *vwa1*-ATG-MO-mCherry plasmid. **(B)** At 1 dpf, fluorescence could be observed in embryos injected with pCS2 + *vwa1*-ATG-MO-mCherry mRNA (a), showing that the mRNA could be translated into mCherry fluorescent protein. No fluorescence was observed in embryos co-injected with pCS2 + *vwa1*-ATG- MO-mCherry mRNA and *vwa1*-ATG MO (b), showing that mCherry was effectively knocked down. Fluorescence similar to that of pCS2 + *vwa1*-ATG-MO-mCherry injected embryos was observed in embryos co-injected with pCS2 + *vwa1*-ATG-MO-mCherry mRNA and *p53* MO (c), showing that unspecific MO could not knock down mCherry.

FIGURE S2 Deformities of pharyngeal cartilage in *vwa1* morphants. The figure shows the jaws of uninjected and *vwa1* morpholino (MO)-, *p53* MO- and *vwa1* + *p53* MO-injected morphants Alcian blue staining of morphant embryos in different dose. Injection of *vwa1*-ATG MO and *vwa1* + p53 MO caused deformities in pharyngeal cartilages, and higher amounts of MO caused more severe deformities. There was no significant difference compared with controls after injection of *p53* MO.

TABLE S1 Sequences of the primers of gRNA and the probes of the markers.
