# Supplementary figures and images for: A Mutation in VWA1, Encoding von Willebrand Factor A Domain-Containing Protein 1, Is Associated With Hemifacial Microsomia
Source: Front Cell Dev Biol. 2020 Sep 9;8:571004. doi: 10.3389/fcell.2020.571004 (PMC7509151; doi:10.3389/fcell.2020.571004)

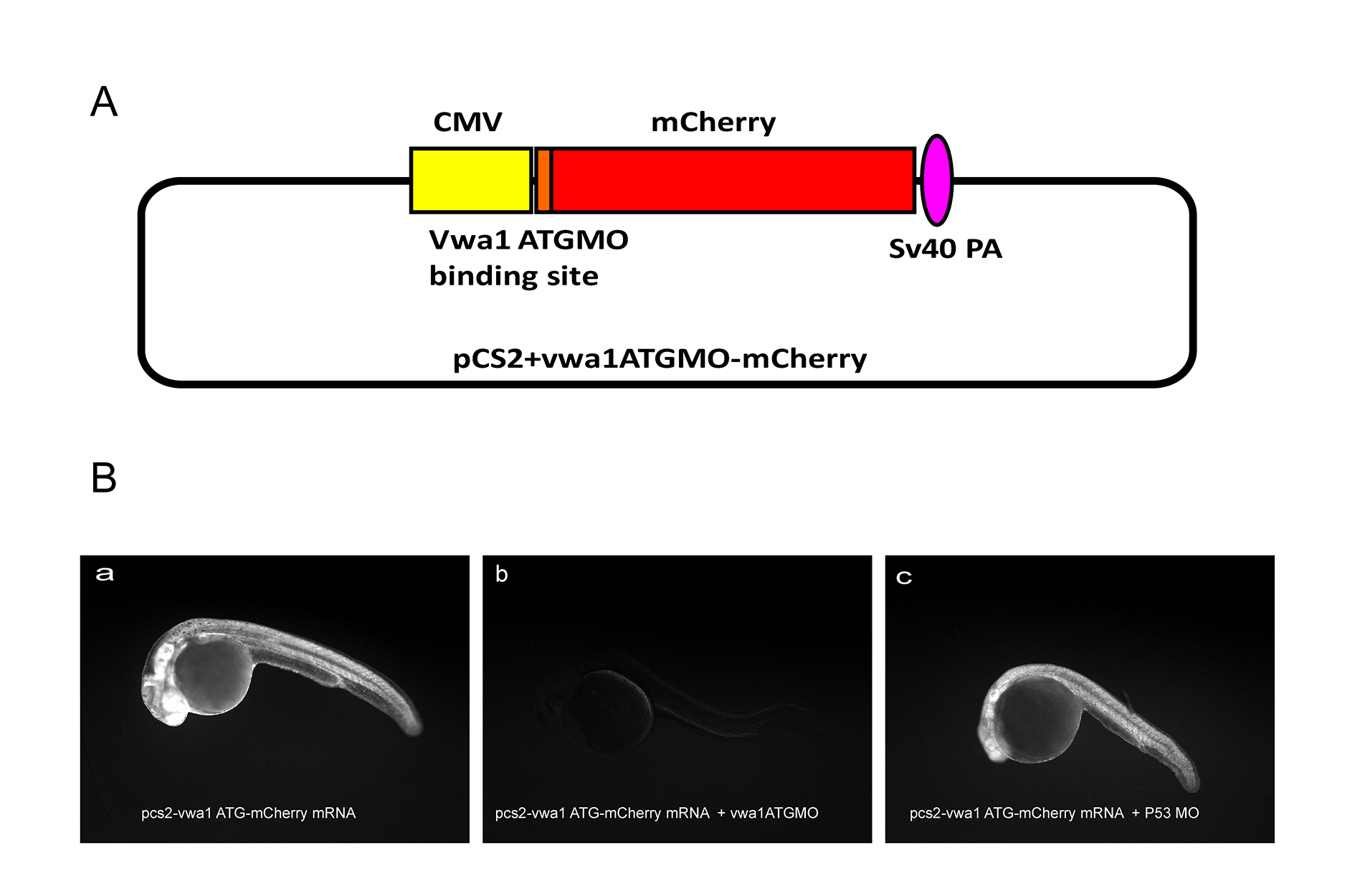

Supplement: FIGURE S1 — Efficacy of vwa1-ATG Morpholino. (A) Structure of pCS2 + vwa1-ATG-MO-mCherry plasmid. (B) At 1 dpf, fluorescence could be observed in embryos injected with pCS2 + vwa1-ATG-MO-mCherry mRNA (a), showing that the mRNA could be translated into mCherry fluorescent protein. No fluorescence was observed in embryos co-injected with pCS2 + vwa1-ATG- MO-mCherry mRNA and vwa1-ATG MO (b), showing that mCherry was effectively knocked down. Fluorescence similar to that of pCS2 + vwa1-ATG-MO-mCherry injected embryos was observed in embryos co-injected with pCS2 + vwa1-ATG-MO-mCherry mRNA and p53 MO (c), showing that unspecific MO could not knock down mCherry. [file Image_1.TIF]

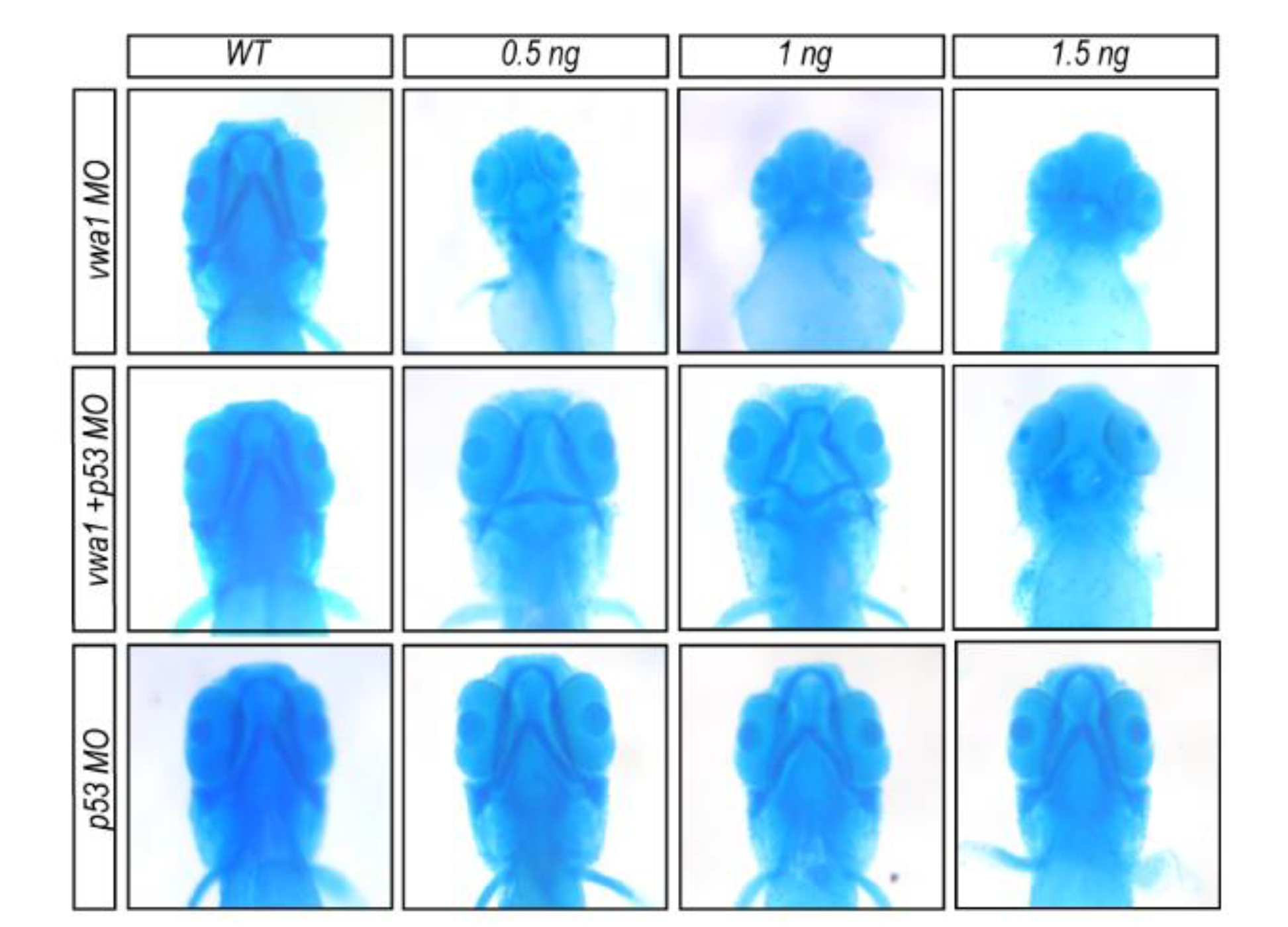

Supplement: FIGURE S2 — Deformities of pharyngeal cartilage in vwa1 morphants. The figure shows the jaws of uninjected and vwa1 morpholino (MO)-, p53 MO- and vwa1 + p53 MO-injected morphants Alcian blue staining of morphant embryos in different dose. Injection of vwa1-ATG MO and vwa1 + p53 MO caused deformities in pharyngeal cartilages, and higher amounts of MO caused more severe deformities. There was no significant difference compared with controls after injection of p53 MO. [file Image_2.TIF]
