# Supplementary material for: A Mutation in VWA1, Encoding von Willebrand Factor A Domain-Containing Protein 1, Is Associated With Hemifacial Microsomia
Source: Front Cell Dev Biol. 2020 Sep 9;8:571004. doi: 10.3389/fcell.2020.571004 (PMC7509151; doi:10.3389/fcell.2020.571004)
Supplement: TABLE S1 — Sequences of the primers of gRNA and the probes of the markers. [file Table_1.DOCX]

**Supplementary Table 1. Sequences of the primers of gRNA and the probes of the markers**

| Primers of gRNA and markers | Sequences（5’-3’） |
| --- | --- |
| *vwa1* gRNA Target1 F | TAATACGACTCACTATAGGCTCACATGATCTGGGCCCGTTTCAGAGCTATGCTGG |
| *vwa1* gRNA Target2 F | TAATACGACTCACTATAGGTCGTGAGTCCGCCTACTGGTTTCAGAGCTATGCTGG |
| *vwa1* gRNA Target3 F | TAATACGACTCACTATAGGGATCTGTCCCGTCCTCATGTTTCAGAGCTATGCTGG |
| *vwa1* gRNA Target4 F | TAATACGACTCACTATAGGCACTCGAAGCGGGCCACTGTTTCAGAGCTATGCTGG |
| *vwa1* gRNA R | AAAAAAAGCACCGACTCGGTGCCAC |
| *dlx2* Probe F | AGCCAAAGAAAGTCCGAAAA |
| *dlx2* Probe R | CCGCAGAGTTCGTGGATGAA |
| *dlx2* Probe B-T7 R | TAATACGACTCACTATAGGGCCGCAGAGTTCGTGGATGAA |
| *sox9a* Probe F | GCAGCAGGGCTCTCCGCAGC |
| *sox9a* Probe R | GCATGCAAATTAAGTAGAAC |
| *sox9a* Probe B-T7 R | TAATACGACTCACTATAGGGGCATGCAAATTAAGTAGAAC |

F, Forward; R, Reverse.
